# Unveiling microRNA-like small RNAs implicated in the initial infection of *Fusarium oxysporum* f. sp. *cubense* through small RNA sequencing

**DOI:** 10.1080/21501203.2024.2345917

**Published:** 2024-05-05

**Authors:** Lifei Xie, Yuntian Bi, Chengcheng He, Junjian Situ, Meng Wang, Guanghui Kong, Pinggen Xi, Zide Jiang, Minhui Li

**Affiliations:** Department of Plant Pathology/Guangdong Province Key Laboratory of Microbial Signals and Disease Control, South China Agricultural University, Guangzhou, China

**Keywords:** Small RNAs, banana Fusarium Wilt, infection process, pathogenicity

## Abstract

Banana Fusarium wilt (BFW), caused by *Fusarium oxysporum* f. sp. *cubense* (*Foc*), poses a major challenge to the worldwide banana industry. Fungal microRNA-like small RNAs (milRNAs) play crucial roles in regulating fungal growth, conidiation, development, and pathogenicity. However, the milRNAs and their functions in the pathogenesis of *Foc* remain poorly understood. In this study, we employed high-throughput sequencing and bioinformatics to profile *Foc* sRNAs during both pure culture and early infection stages. Our analysis identified six milRNAs exhibiting significantly upregulated expression at the initial *Foc* infection. Of these, milR106’s biogenesis was found to be Dicer-dependent, whereas milR87, milR133, milR138, and milR148 were associated with Dicer and Argonaute proteins. Genetic manipulation and phenotype analysis confirmed that milR106 is crucial for *Foc* virulence by regulating conidiation, hydrogen peroxide sensitivity, and infective growth. Gene Ontology analysis of milRNA targets in the banana genome revealed enrichment in defence response to fungus and cellular response to hypoxia, implying the importance of these target genes in response to pathogen infection. In conclusion, our sRNA profiling of *Foc* identified several infection-induced milRNAs. The corresponding results provide valuable molecular targets for the development of an efficient strategy to control BFW.

## 1. Introduction

*Fusarium oxysporum* f. sp. *cubense* (*Foc*) is a typical soil-borne fungus that causes *Fusarium* wilt by infecting the roots and blocking the vascular tissues of the host banana and threatens global banana production (Viljoen et al. 2020). A total of four races have been reported in *Foc*, of which the tropical race 4 (TR4) is the most widespread. Banana Fusarium Wilt (BFW) caused by TR4 is the most devastating disease in banana-producing countries and regions (Zhang et al. 2019). It is well known that the most effective method to control BFW is implying strict phytosanitary measures and planting disease-resistant cultivars (Dita et al. 2018). However, breeding disease-resistant cultivars that simultaneously meet market standards while maintaining high yields is challenging. Additionally, these disease control measures are very difficult to enforce by smallholder growers (Viljoen et al. 2020). In some severely affected banana plantations, the conventional ‘Cavendish cultivar had to be abandoned for other alternative crops due to the spread of TR4 (Warman and Aitken 2018). Therefore, a comprehensive understanding of the pathogenesis of BFW and the development of improved control methods are urgently needed.

MicroRNA-like small RNAs (milRNAs), first found in *Neurospora crassa* and *Botrytis cinerea*, resemble plant and animal microRNAs and play crucial roles in post-transcriptional regulation of gene expression (Lee et al. 2010; Weiberg et al. 2013; Jin et al. 2018). These milRNAs, typically 19–25 nt in length, derive from single-stranded RNA transcripts with stem-loop structures and silence complementary transcripts through RNA-mediated interference (RNAi) (Bartel 2004; Nicolás and Ruiz-Vázquez 2013). A growing number of milRNAs have been recognised for their roles in fungal growth and development, such as those involved in sclerotia development in *Sclerotinia sclerotiorum* (Xia et al. 2020), vegetative form of mycelial or yeast phases of dimorphic fungus *Penicillium marneffei* (Lau et al. 2013), and spore morphological differences of *Metarhizium acridum* (Zhang et al. 2022). Recent studies reported that milRNAs secreted by phytopathogenic fungi through extracellular vesicles play important roles in plant-microbe interaction (Huang et al. 2019; He et al. 2023). For instance, milRNAs of *B. cinerea* have been identified as small RNA effectors that suppress host immunity and facilitate fungal infection through cross-kingdom RNAi (Weiberg et al. 2013; Wang et al. 2017). Similarly, the *Puccinia striiformis* f. sp. *tritici* (Pst) miRNA, Pst-milR1, promotes plant disease susceptibility by cleaving the *PR2* gene (Wang et al. 2017). The milRNA Vm-milR37 exclusively expressed in the mycelium, was found to contribute to pathogenicity in *Valsa mali* (Feng et al. 2021).

Although the regulatory role of endogenous milRNAs in pathogenicity has been characterised in some fungi, such as *B. cinerea*, *P. striiformis*, and *Va. mali* (Weiberg et al. 2013; Wang et al. 2017; Feng et al. 2021). Little is known about the role of small RNAs produced by the soil-borne fungus *Foc* in pathogenicity and other biological processes. In this study, we conducted high-throughput sequencing and bioinformatics analysis to profile the sRNAs of *Foc* during both pure culture and early infection stages. We aimed to identify the milRNAs that were significantly induced during the initial infection process of *Foc*, laying the foundation for further elucidation of the pathogenic mechanism of *Foc*. We confirmed that one of the milRNAs is Dicer-dependent and involved in the pathogenicity of *Foc*. The results provide valuable molecular targets for the development of efficient strategies to control BFW and breed disease-resistant cultivars.

## 2. Materials and methods

### 2.1. Fungal strains and sample preparation for small RNA sequencing

The wild-type (WT) strain XJZ2 of *F. oxysporum* f. sp. *cubense* tropical race 4 (Li et al. 2013) was activated on PDA plates at 28 °C and then transferred to YPD media to prepare the conidia suspension. The triploid cultivated banana cultivar ‘Canvendish’, widely grown in production (Li et al. 2024), was used for infection inoculation following the previously described method (Li et al. 2014). Briefly, healthy banana roots were immersed in the suspension with a conidia concentration of 1 × 10^8^ conidia/mL. After 36 h of inoculation, banana roots with attached *Foc* conidia and mycelia were collected for total RNA extraction. The same concentration of conidia was inoculated in a minimal medium (MM) and cultured for 36 h to collect pure cultured conidia and mycelia as a control. Each sample was prepared in triplicate.

### 2.2. RNA extraction and sRNA sequencing

200 mg of banana roots or *Foc* mycelia from different treatments were collected and ground into powder using liquid nitrogen. Total RNA extraction was performed using a CTAB reagent (De Wever et al. 2020). RNA integrity was assessed and quantified with a Bioanalyzer 2100 (Agilent, Santa Clara, USA), and only qualified RNA samples with a RIN (RNA Integrity Number) value between 7 and 10 were sent to Novogene (Beijing, China) for sRNA library construction and high-throughput sequencing (Ouyang et al. 2021).

### 2.3. Sequence data analysis and infection-induced milRNA prediction

Raw sequencing data were trimmed and analysed using CLC Genomics Workbench v11 (CLC bio) following our previous description (Li et al. 2022). Only reads mapped to the *Foc* II5 (TR4 strain) genome were used for data analysis. To minimise false positive sRNAs induced by mRNA degradation only reads mapped to the UTR, intron, and intergenic regions were used for sRNA length distribution and sRNA locus identification. The RNAstructure (Version 6.3) was employed to predict the secondary structure of the sRNA precursor (Reuter and Mathews 2010).

To identify differentially expressed sRNAs and their loci reads from the pure culture stage and initial infection stage of *Foc* were compared. To assess sRNA expression, read counts of more than 10 were normalised to transcripts per million (TPM) values, and log2 ratios were calculated using the TPM value from the samples of XJZ2-36 hpi and XJZ2. Those sRNAs, that were significantly up-regulated [log_2_^TPM (XJZ2–36 hpi/XJZ2)^ >1, *p* < 0.05] in the infection stage compared to the pure culture stage, along with their precursors exhibiting a typical secondary hairpin structure, were considered as infection-induced milRNAs (Lee et al. 2010; Li et al. 2022).

### 2.4. Validation of milRNA by qRT‑PCR

To validate the predicted and differentially expressed milRNAs, quantitative reverse transcription-PCR (qRT-PCR) was performed according to our previously described method (Li et al. 2022). The milRNA was first added to a PolyA tail, and then the cDNA was reverse-transcribed using an oligo-dT adaptor. Subsequently, the expression of milRNAs was quantified by qRT-PCR. At least three biological replicates were performed for each sample.

### 2.5. Generation and validation of the milRNA deletion mutants and the complemented strains

The precursor sequence (approximately 300 bp) of the milRNA was replaced and complemented through homologous recombination through PEG-mediated protoplast transformation (Li et al. 2014). The milRNA precursor was first introduced into the Geneticin resistance gene cassette of pKNT-G418 and then transformed after ligating it with its left and right border sequences, respectively. The milRNA deletion mutants and the complemented strains were identified by PCR using the primers provided in Table S1.

### 2.6. Fungal invasion assays and pathogenicity test

The ability of mycelia to penetrate cellophane and their invasive growth on tomato fruit surfaces were compared between the WT and the mutants according to the previously described method (Li et al. 2014). Pathogenicity on banana plantlets was assessed as described previously (Li et al. 2013). Each treatment involved the use of 30 banana plantlets, and the incidence and disease indexes were calculated for disease severity assessment (Li et al. 2013).

### 2.7. MilRNA target gene prediction and functional annotation

The potential target genes of the milRNAs were predicted using the psRNATarget algorithm (https://www.zhaolab.org/psRNATarget/analysis) with default parameters (Li et al. 2022). The Gene Ontology (GO) pathway classification of target genes from the *Foc* genome was implemented on the NovoMagic cloud analysis platform (Novogene, China). For annotation of target genes derived from the banana genome (Ahmad et al. 2020), the website of Banana Genome Hub (https://banana-genome-hub.southgreen.fr/content/go-enrichment) was utilised for GO enrichment analysis, with a highly sensitive parameter to merge similar GO terms.

### 2.8. Statistical analysis

In the study, a Student’s *t*-test was used for the significance analysis of two samples. A Duncan’s multiple range test was used to analyse the data of multiple samples at both levels (α = 0.05 and α = 0.01). And different letters on the bars indicated significant differences.

## 3. Results

### 3.1. *Sequence analysis of sRNAs in* Foc *during pure culture and initial infection stages*

In this study, the small RNAs of *F. oxysporum* f. sp. *cubense* in the pure cultured stage (Culture) and the initial infection stage (36 h post-inoculation, 36 hpi) of banana roots were sequenced respectively. After the elimination of the 5’ adaptor, contamination, and low-quality sequences from the raw data, reads with lengths ranging from 16 to 40 nucleotides (nt) were preserved. For subsequent sRNA analysis, only reads that perfectly matched the *Foc* II5 genome were used. After data processing, we obtained over 16 million high-quality small RNA sequences, with 9,221,095 reads from the pure cultured stage and 6,899,403 reads from the initial infection stage (Table S2). Each sample was replicated three times, and each replicate contained more than two million reads, providing sufficient data for subsequent analysis. Less than 7% of the sequences from *Foc*-36 hpi could be mapped to the genome, compared to an average of 27% of the sequences from *Foc*-culture (Table S2). This discrepancy may be attributed to the substantial number of sequences derived from the host banana in the *Foc*-36 hpi data, in contrast to the pure culture data of *Foc*.

**Figure 1.**
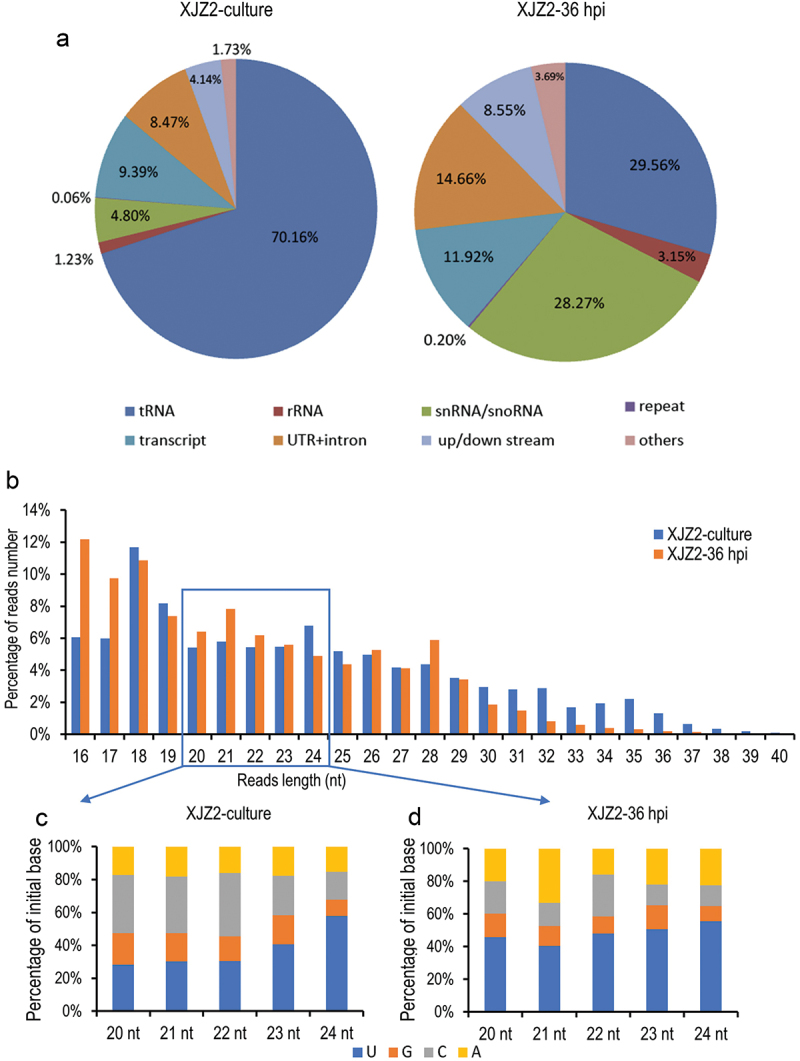
Small RNA sequencing analysis in *Fusarium oxysporum* f. sp. *cubense* (*Foc*). (a) Classification and annotation of sRNAs. (b) sRNA length distribution. (c) and (d) Statistical analysis of initial bases in sRNA from XJZ2-culture (c) and XJZ2-36 hpi (d).

All sRNA reads mapped to the *Foc* II5 genome were aligned to the data sets of the Rfam database, including rRNA, tRNA, snRNA, snoRNA, and repeat elements, as well as transcripts, genes, and the upstream and downstream regions (1,000 bp before and after the gene sequence) in turn. Very few small RNAs without annotation were found in the two samples. In the pure cultured stage, the majority of reads (76.25%) were mapped to the Rfam database. Among them, most of the reads (70.16%) were aligned to tRNA. However, in the infection stage (36 hpi), approximately 61% of the reads were aligned to the Rfam database, with only 29.56% derived from tRNA. Notably, the reads from rRNA and snRNA/snoRNA showed a significant increase in the 36 hpi sample compared to the pure cultured sample (Figure 1a). The ratios of reads mapped to introns, 3’/5’ UTRs, as well as intergenic regions (upstream and downstream of genes) were significantly higher in the infection stage, compared to the pure cultured stage (Figure 1a). However, there was no significant difference in the proportion of reads mapped to transcripts between the two samples. After sequence alignment, reads that were mapped to the Rfam database and transcripts were excluded, and the remaining reads from introns, 3’/5’ UTRs, and intergenic regions were used for further sRNA data analysis.

The lengths of the reads were statistically analysed, and the results showed that approximately 58% of the sRNA reads were 18–26 nt in length in both samples (Figure 1b). Among these, the most frequently sequenced reads were 18 nt in length, followed by 19 nt and 24 nt in the pure cultured stage. In the infection stage of 36 hpi, 16-nt reads were the most abundant, followed by 18-nt and 21-nt reads.

Considering that the reported size of miRNA in Eukaryotes is typically between 20 and 24 nt (Bartel 2004; Lee et al. 2010), reads with lengths ranging from 20 to 24 nt were used to analyse the bias in the initial nucleotide. In both samples, the majority of sRNAs started with U. Reads starting with U averagely accounted for 37.5%, followed by reads starting with C and A, with average proportions of 29.82% and 16.87%, respectively, while sRNAs starting with G were the least abundant in the pure cultured *Foc* (Figure 1c). In the infection stage of 36 hpi, sRNAs starting with U remained the most abundant, accounting for an average of 48.15%, followed by sRNAs starting with A and C, accounting for 22.72% and 17.01%, respectively, while those starting with G were the least abundant (Figure 1d). Compared to the pure cultured *Foc*, the reads starting with U and A were significantly increased upon inoculation of the host plant, indicating that sRNAs with a bias towards U and A as the initial nucleotides may play important roles in infection processes.

### 3.2. *Screening and identification of induced milRNAs in* Foc *during the initial infection stage*

To identify milRNAs in *Foc*, unannotated reads were blasted against the miRbase (Release 21.0). Only a few reads were mapped to known miRNAs in the database, and no difference in the expression of miRNAs was detected between the two samples. Consequently, we failed to predict any known milRNA in *Foc*, which is consistent with the absence of miRNA orthologous between filamentous fungi and other eukaryotic species (Chen et al. 2014).

**Figure 2.**
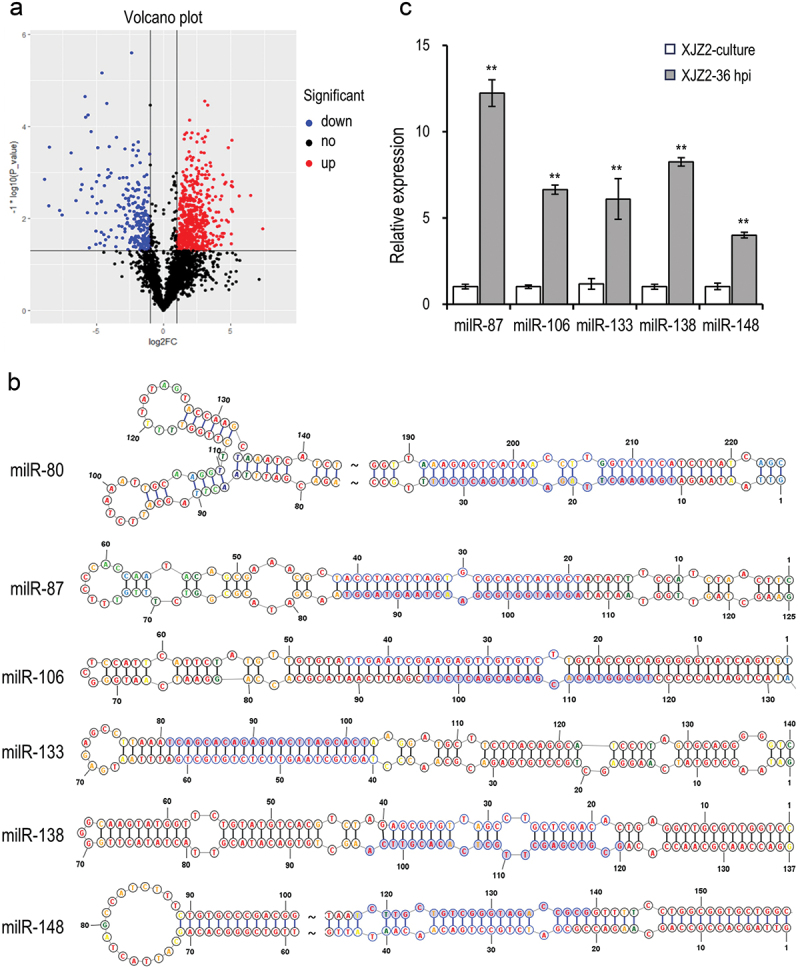
Identification of infection-induced milRNAs in *Fusarium oxysporum* f. sp. *cubense*. (a) Differential expression analysis of sRNAs of *Foc* in the pure culture stage and the initial infection stage. (b) Precursors’ secondary structure and sequences of infection-induced milRNAs in *Foc*. The mature milRNA sequences were marked in light blue. (c) Expression of the milRNAs in pure culture stage and initial infection stage by qRT-PCR. A Student’s *t*-test was used for significant analysis. **, *p* < 0.01. Error bars indicate S.D. (*n* = 3).

To identify infection-induced milRNAs in *Foc*, we determined the sRNAs-producing sites and screened the precursors with hairpin structures. Based on normalization by calculating the transcripts per million (TPM) value of each sRNA-producing locus, the expression levels of sRNAs were compared between the two samples. A total of 3,439 sRNAs-producing loci were captured. Among them, 953 and 327 sRNAs showed significant up- and down-regulated expression, respectively, in the infection stage compared to the pure-cultured stage (Figure 2a). After predicting the secondary structure of sRNA precursors and verifying their expression, we identified six loci of infection-induced milRNAs with hairpin structures (Figure 2b). These milRNAs were primarily derived from intergenic regions, except for milR106, which was from an intron. The results of qRT-PCR confirmed that the expression of the milRNAs was consistent with sequencing results when using small nuclear RNA U4 of *Foc* as the internal control (Figure 2c). However, one milRNA (milR-80) did not show specific products for its low GC content.

To validate the identified milRNA sequences in *Foc*, we amplified the milRNAs using small RNA reverse transcription products as templates. Subsequently, the PCR products were cloned into a T vector for sequencing. The resequencing results of fungal milRNA clones (Data not shown) confirmed that the mature milRNA sequences were consistent with the original high-throughput sequencing results and with a length ranging from 21 to 24 nt (Figure 2b). These findings provide additional evidence that the six small RNAs obtained through our screening process met the criteria for fungal milRNAs and were recognised as *Foc*_milRNAs induced by infection.

### 3.3. *Identification of infection-induced milRNAs biosynthetic pathways in* Foc

**Figure 3.**
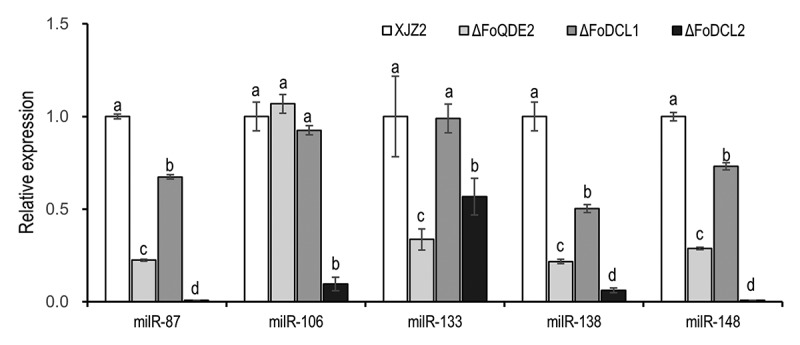
Expression of the milRNAs in different gene deletion mutants involved in the sRNA biogenesis pathway in *Fusarium oxysporum* f. sp. *cubense*. Transcriptional levels of the infection-induced milRNAs were detected by qRT-PCR. A Duncan’s multiple range test was used for significant analysis. Error bars indicate S.D. (*n* = 3). Different letters on the bars indicate significant differences at the level of α = 0.01.

Argonaute (AGO) and Dicer proteins are known to play key roles in milRNA production in fungi (Lee et al. 2010). The expression of infection-induced milRNAs was detected by qRT-PCR in previously screened Δ*FoQDE2*, Δ*FoDCL1*, and Δ*FoDCL2* mutants (Li et al. 2022), as well as WT strain. And the biosynthetic pathways of the milRNAs were analysed according to their expression levels. The results showed that the expression levels of milR87, milR133, milR138, and milR148 were significantly decreased in both Δ*FoQDE2* and Δ*FoDCL2* mutants compared to the WT, indicating the biosynthesis of these four milRNAs is dependent on the *FoDCL2* and *FoQDE2* genes. On the other hand, milR106 expression was significantly decreased only in Δ*FoDCL2* mutants, suggesting that its biosynthesis is solely dependent on the *FoDCL2* gene (Figure 3).

### 3.4. *Deletion and complementation of milR106 in* Foc

**Figure 4.**
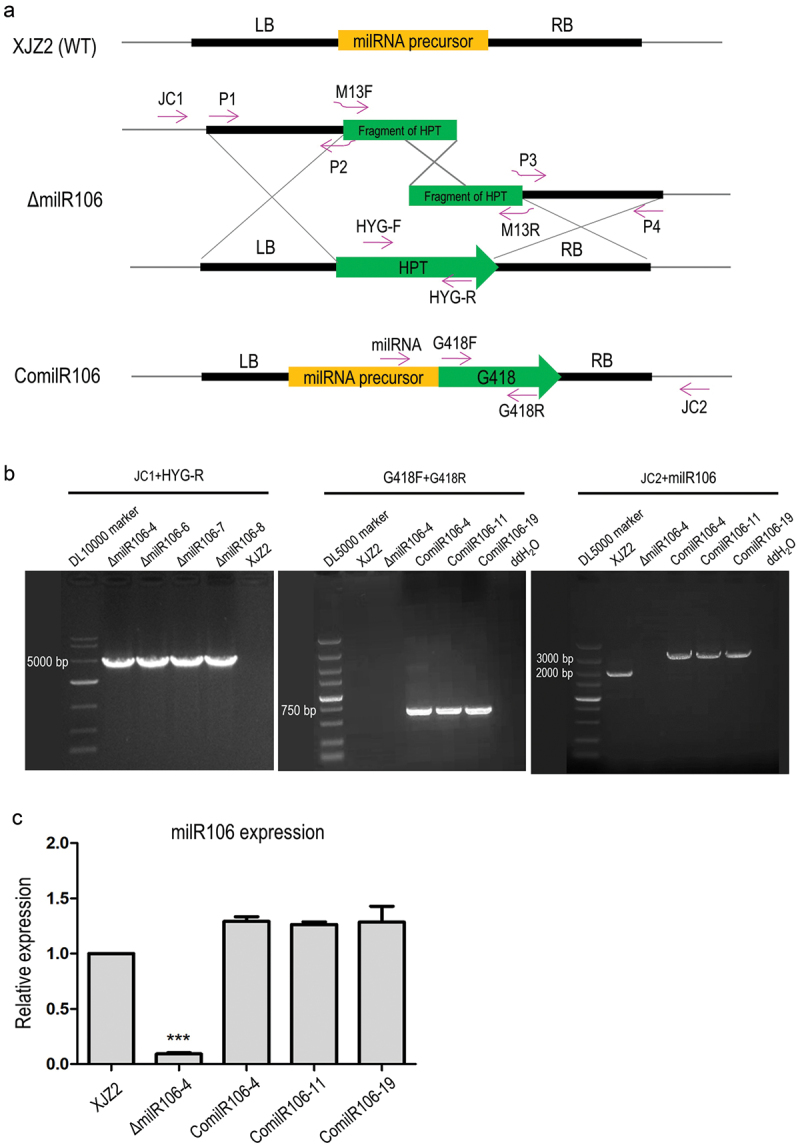
Verification of the milR106 deletion and complementation mutants by PCR and qRT-PCR. (a) Schematic diagram for deletion and complementation of milR106 in *Foc*. (b) PCR identification of the milR106 deletion mutants and complemented strains. (c) Transcriptional level of milR106 in different mutants of *Foc* was detected by qRT-PCR. To assess the statistical significance between the wild-type strain XJZ2 and the Δ*milR106-4* mutant, a Student’s *t*-test was used. ***, *p* < 0.001. Error bars indicate S.D. (*n* = 3).

Among the six infection-induced milRNAs identified, milR106 is exclusively dependent on the *FoDCL2* gene for its biogenesis. These unique biogenesis characteristics and the expression pattern of upregulation during the early infection stages prompted us to select milR106 for comprehensive functional investigation. To investigate the function of milR106 in *Foc*, we generated the milRNA precursor deletion mutants from the WT strain XJZ2 via homologous recombination. Additionally, we created complementation transformants based on one of the deletion mutants Δ*milR106-4* (Figure 4a). Four milRNA-deleted mutants were confirmed by PCR with the primer JC1 from the 5’ flanking region and primer HYG-R located in the hygromycin resistance gene (HYG) (Figure 4b). Using the primer pair G418F/G418R designed from the geneticin resistance gene, an amplicon was observed in the complemented transformants (ComilR106-4/-11/-19), but not in the WT strain, Δ*milR106* mutant or the negative control (ddH_2_O) (Figure 4b). The three complemented transformants also showed an amplicon when PCR amplification was performed with primer milR106 combined with primer JC2 from 3’ flanking region as shown in Figure 4b, indicating successful complementation of milR106 in the Δ*milR106* deletion mutant. This result was further confirmed by qRT-PCR (Figure 4c).

### 3.5. *MilR106 regulates tolerance to hydrogen peroxide and is involved in the virulence of* Foc

**Figure 5.**
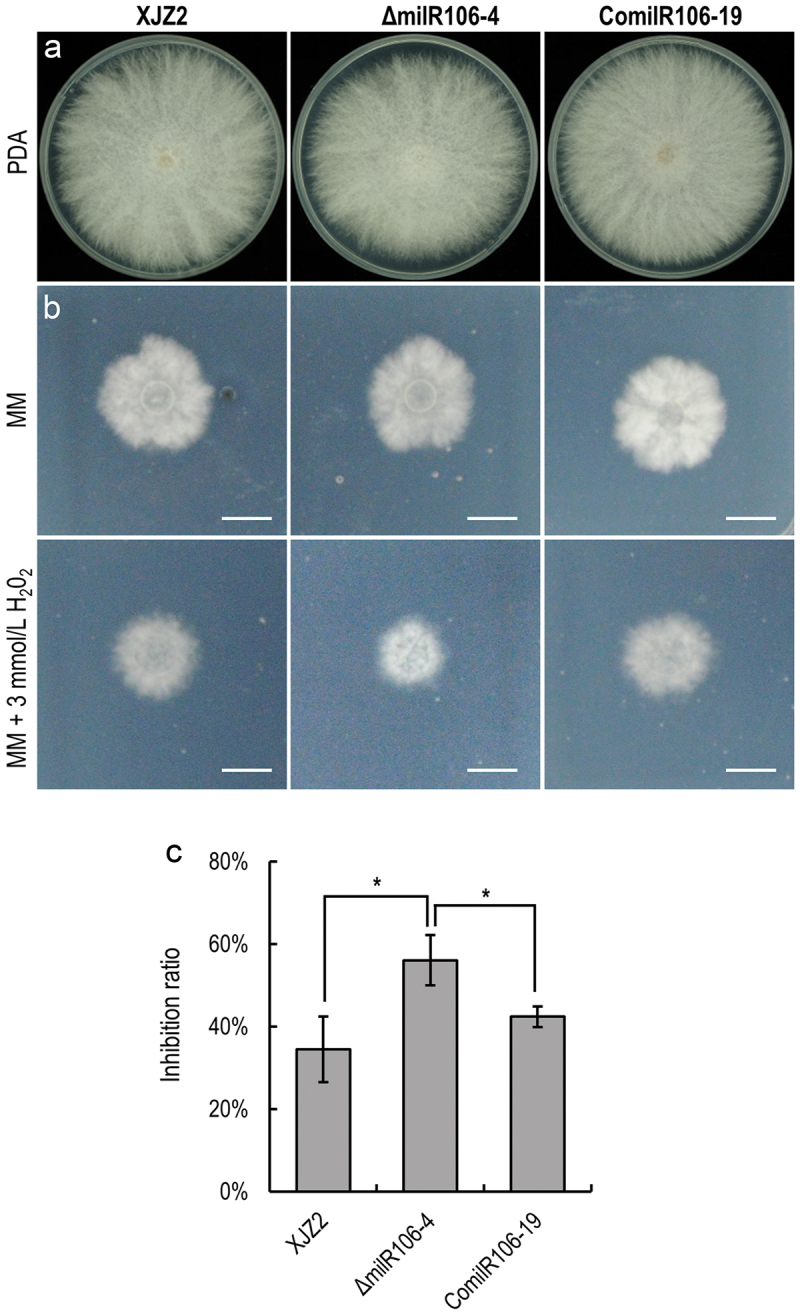
Characterization of milR106-deleted mutant and complemented strains in *Fusarium oxysporum* f. sp. *cubense*. (a) Colony morphology of the WT strain XJZ2, the Δ*milR106* mutant (Δ*milR106-4*), and milR106 complemented strain ComilR106-19 on PDA plates. (b) Mycelial sensitivity to H_2_O_2_. Mycelial growth of the above strains was measured at MM and MM with 3 mmol/L H_2_O_2_. Bar=1 cm. (c) Percentage of mycelial growth inhibition by H_2_O_2_. A Student’s *t*-test was used for significant analysis. *, *p* < 0.05. Error bars indicate S.D. (*n* = 3).

To decipher the biological functions of milR106 in *Foc*, we assessed the colony growth and conidiation in the WT strain and the mutants. The WT and the milR106 complemented strains exhibited normal growth on PDA (Figure 5a) and minimal medium (MM) (Figure 5b). The Δ*milR106* mutant showed a slight attenuated growth on PDA, although statistical analysis revealed no significant difference compared to the WT strain (Figure 5c). The Δ*milR106* mutant produced much fewer microconidia than the WT and the complemented transformants after 7 days of culture on PDA (Figure S1). Moreover, the Δ*milR106* mutant displayed hypersensitive to oxidative stress when cultured on MM supplemented with 3 mmol/L H_2_O_2_ (Figure 5b,c). In contrast, the growth of the milR106 complemented strains was unaffected by oxidative stress (Figure 5b,c). These results indicate that milR106 positively regulates tolerance to oxidative stress in *Foc*.

**Figure 6.**
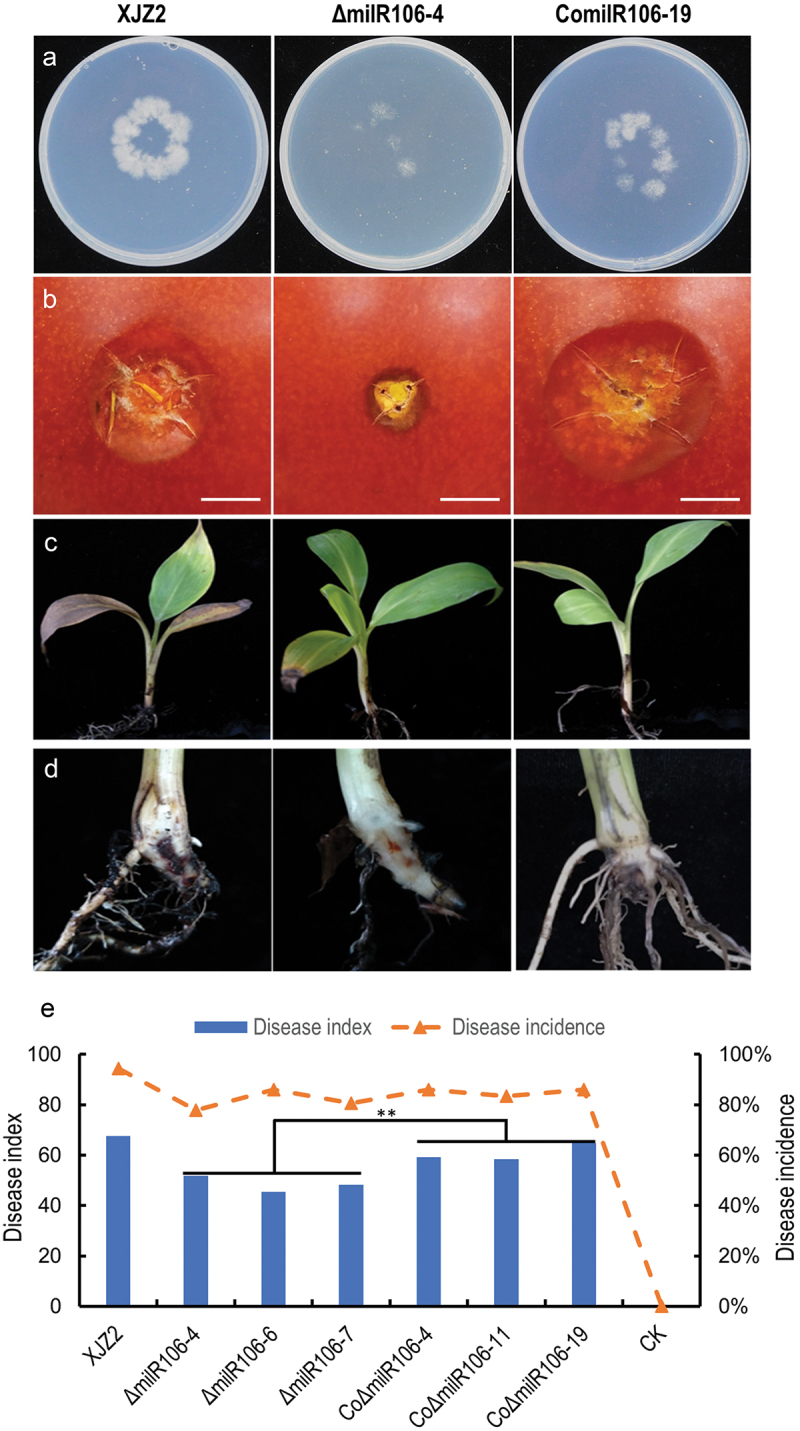
Assessment of invasive growth and Pathogenicity. (a) Cellophane penetration assay comparing the invasive growth of the WT strain XJZ2, Δ*milR106-4* mutant, and the complemented strain ComilR106-19. Conidia suspensions (100 μL per strain) with the same concentration of 1 × 10^5^ spores/mL were put on cellophane-covered PDA plates and incubated at 28 °C for 4 days, then the cellophane sheets were removed, and samples were incubated for an additional 3 days and the colony size indicated mycelial growth were photographed. (b) Invasive growth on tomato fruits. The surfaces of tomato fruits were inoculated with discs of the different strains. The symptoms of necrosis on tomato fruits were recorded at 5 days post-inoculation. Bar =1 cm. (c) and (d) Symptoms of pathogenicity on the leaves (c) and corms (d) of the banana plantlets. (e) Disease index and disease incidence of banana plantlets after inoculation with the different strains. A Student’s *t*-test was used for significant analysis. **, *p* < 0.01.

Pathogenicity tests showed that the Δ*milR106* mutant exhibited a compromised ability to penetrate the cellophane membrane (Figure 6a) and caused smaller necrosis areas on the surface of tomato fruits than the WT (Figure 6b). Conversely, the complemented transformant showed the opposite phenotype (Figure 6a,b). In the banana seedling infection assay, Δ*milR106* showed significantly reduced virulence compared to the WT (Figure 6c–e). However, the virulence of the complemented transformants was significantly enhanced, as evidenced by the presence of obvious internal disease symptoms of brown discoloration and higher disease indices (Figure 6d,e). These findings suggest that milR106 contributes to the infective growth and virulence of *Foc*.

### 3.6. *Prediction and GO enrichment of target genes of infection-induced milRNAs in* Foc

To investigate the significance of milRNAs in the *Foc* infection process, we predicted the target genes of milRNAs that expression was significantly induced in the initial infection stage. Using psRNATarget online software, we predicted a total of 296 unigenes from the *Foc* II5 genome and 1,256 unigenes from the banana genome (*M. acuminata* DH Pahang v4.3) as targets of the infection-induced milRNAs. In the banana genome, some *Foc*-milRNAs could target the same gene, such as milR106 and milR87, they both targeted one gene of Macma4_06_g01500. Additionally, some milRNAs targeted a single gene but had multiple target sites, such as milR133, milR148, and milR80. Multiple target sites were more common in the *Foc* genome.

**Figure 7.**
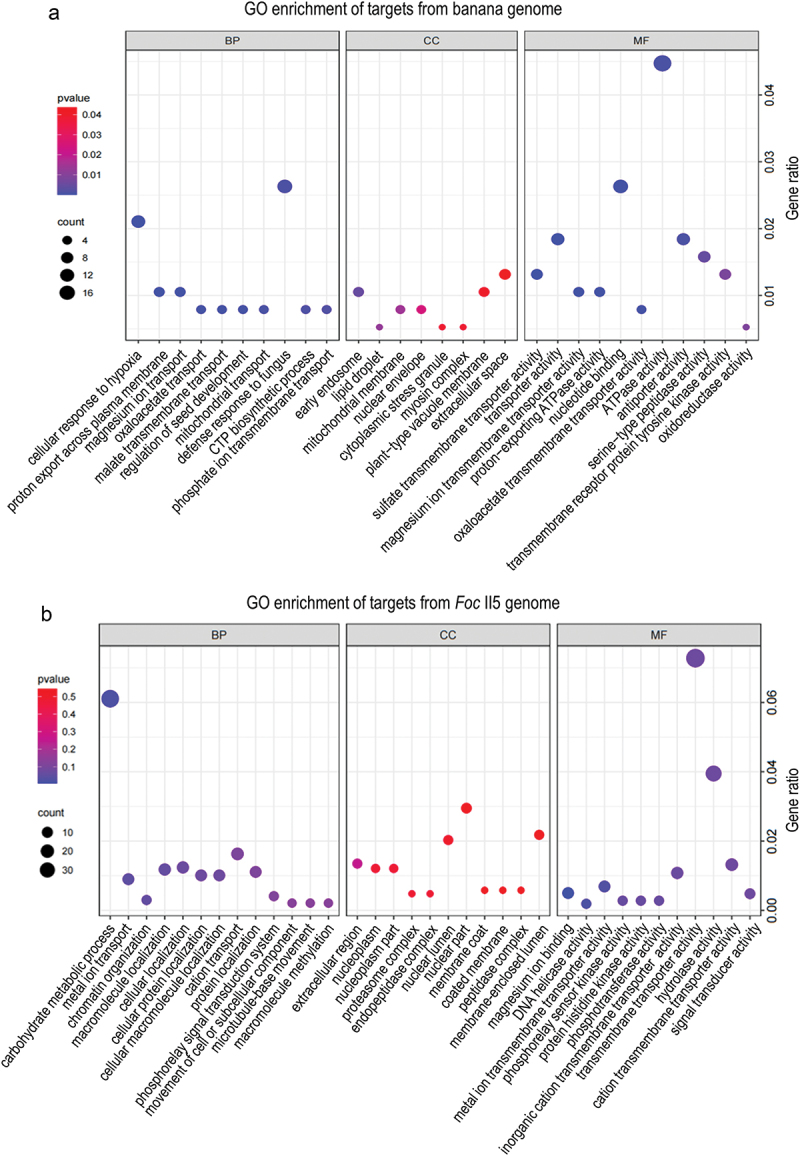
GO enrichment of target genes of six infection-induced milRNAs in *Foc*. (a) Target genes from the host banana genome. (b) Target genes from the *Foc* II5 genome.

GO enrichment analysis of biological processes demonstrated that GO terms such as defence response to fungus (GO:0050832) and cellular response to hypoxia (GO:0071456) were exclusively enriched among the target genes from the banana genome (Figure 7a), suggesting that these infection-induced milRNAs are more likely to target resistant genes in host plants. Furthermore, a lot of genes involved in proteolysis (GO:0006508) and response to oxidative stress (GO:0006979) were also enriched (Figure 7a). The most prevalent GO terms for molecular functions were ATPase activity (GO:0016887), nucleotide binding (GO:0000166), and transporter activity (GO:0005215). As for cellular components, the GO term early endosome (GO: 0005769) was uniquely enriched (Figure 7a).

GO term analysis of target genes in *Foc* showed significant enrichment in biological processes such as methylation (GO:0032259), carbohydrate metabolic process (GO:0005975), and metal ion transport (GO:0030001) (Figure 7b). For molecular functions, a large number of genes engaged in transmembrane transporter activity (GO:0022857) and hydrolase activity (GO:0004553) were enriched. However, there was no significant GO terms enrichment for cellular components (Figure 7b). The diverse functions of these target genes indicate that milRNAs have crucial roles in regulating gene expression in a wide range of biological processes, particularly in plant defence responses to pathogen infections.

In this study, knockout of milR106 in *Foc* results in increased sensitivity to hydrogen peroxide and decreased virulence, suggesting a potential involvement of the milRNA in hydrogen peroxide clearance, reactive oxygen species (ROS) homoeostasis regulation, antioxidant defence, and response to oxidative stress. To elucidate the mechanism underlying the phenotype, we checked the target genes of milR106 in both pathogen and host plants. Three of the target genes (Macma4_04_g05490, Macma4_06_g02360, and Macma4_02_g06450) encoding peroxidases in banana were enriched in the response to oxidative stress (GO:0006979) (Table S3). The target genes in *Foc* were not enriched in any GO terms related to oxidative stress (Table S4).

## 4. Discussion

milRNAs involved in plant-pathogen interaction have been discovered in some pathogenic fungi by the application of high-throughput sequencing techniques and bioinformatics analysis (Wang et al. 2017; Jin et al. 2019; Feng et al. 2021). In this study, we identified six infection-induced milRNAs by profiling the sRNAs of *Foc* during pure culture and early infection stages. These milRNAs, like other milRNAs identified in *N. crossa* (Lee et al. 2010) and *F. oxysporum* (Chen et al. 2014), lack sequence conservation. Their producing sites are mainly located in the intergenic and untranslated regions (3’/5’ UTR and intron). The findings contrast with those of sRNAs discovered in *B. cinerea* and *S. sclerotiorum*, where the sRNAs are primarily derived from transposable elements (Weiberg et al. 2013; Derbyshire et al. 2019). This discrepancy may be attributed to our sequencing data analysis of the elimination of reads mapped to repetitive elements.

In fungi, at least five types of milRNA biogenesis pathways have been reported (Lee et al. 2010; Jin et al. 2019). Among them, *N. crossa* milR-3 biogenesis is similar to that of plants, which require only Dicer to produce mature milRNA (Jones-Rhoades et al. 2006; Lee et al. 2010). The milR-4 is derived from tRNA, and its production is partially dependent on Dicer. The biogenesis of milR-2 requires AGO protein QDE2 and its catalytic activity but is completely independent of Dicer. For *milR-1*, the most abundant milRNAs in *N. crossa*, its production requires Dicer, QDE2, QIP, and MRPL3 (Lee et al. 2010; Chang et al. 2012). Our data showed that the transcript level of milR106 was considerably lower in the ∆*FoDCL2* mutant but not in the Δ*FoQDE2* and ∆*FoDCL1* mutants when compared to the WT. The result indicates that milR106 is a Dicer-dependent milRNA of the milR-3 type. Whereas the transcript levels of the other three milRNAs, milR133, milR138, and milR148, were significantly decreased in both the Δ*FoQDE2* and ∆*FoDCL2* mutants, like the expression pattern of milR87, which has been identified as a milRNA of the milR-1 type (Li et al. 2022).

Increasing evidence indicates that milRNAs produced by plant pathogenic fungi play a crucial regulatory role in developmental processes and pathogenicity (Wang et al. 2015; Huang et al. 2019). For instance, a novel milRNA (VdmilR1) was identified to accumulate during the late stage of *V. dahliae* infection and was found to regulate pathogenicity by targeting a virulence gene from the fungus itself (Jin et al. 2019). In *V. mali*, the expression of Vm-milR37 was observed in the mycelium, but not in the stage of the infection process. Overexpression of this milRNA did not affect vegetative growth, but significantly decreased pathogenicity (Feng et al. 2021). Here, we identified six milRNAs that were dramatically induced in the early stage of *Foc* infection (36 hpi). Through deletion and complementation of the precursor gene, we confirmed that milR106 is involved in the infective growth and virulence of *Foc*. Our previous study also found milR87 contributes to *Foc* virulence during the early infection stage by silencing a GH79 glycosyl hydrolase, which could activate the general host defence response (Li et al. 2022). These investigations on the function of milRNAs in various fungi reveal that milRNAs expressed at different stages of infection and vegetative growth employ diverse mechanisms to regulate pathogen pathogenicity.

Recently, small RNAs were reported to function by trafficking between the host plants and the fungal phytopathogens and play significant role in the nature of the infection (Weiberg and Jin 2015; Hua et al. 2018; Mahanty et al. 2023). In this study, milR106 and milR87 both targeted Macma4_06_g01500, a gene encoding a pentatricopeptide repeat-containing (PPR) protein. PPR proteins have been demonstrated to participate not only in plant growth and development regulation but also in coping with various biotic and abiotic stresses (Liu et al. 2010; Xing et al. 2018). For instance, PPR proteins found in potatoes are significantly induced in response to the infection of *Ralstonia solanacearum*, a bacterial wilt pathogen, suggesting their involvement in disease resistance (Park et al. 2016).

GO enrichment analysis of target genes from the host plants and the pathogens will help to annotate the function of infection-induced milRNAs. In our study, GO terms of defence response to fungus and cellular response to hypoxia were enriched. Hypoxia stress can be caused by submergence or pathogen infection. These two stresses often occur sequentially or at the same time in nature. Therefore, plants have evolved economical and efficient strategies to deal with the stresses, such as responding to both stresses via a single signalling pathway (Tang and Liu 2021; Valeri et al. 2021). Although no significant enrichment of GO terms related to oxidative stress was observed among the target genes of milR106 in the pathogen, three peroxidase-encoding genes in the host banana were enriched in the GO term “response to oxidative stress” (GO:0006979). Peroxidases, as integral components of antioxidant defence systems, play a crucial regulatory role in maintaining ROS homoeostasis and supporting cellular functions, such as antioxidant defence and plant defence responses, by modulating ROS levels (Daudi et al. 2012). Our finding revealed that milR106 targets several genes encoding peroxidases. Therefore, it is not difficult to understand that infection-induced milR106 may target and suppress plant peroxidase expression, inhibiting plant defence responses such as hypersensitive reaction response and ROS burst, thereby facilitating pathogen infection. In contrast, the loss of milR106 makes the pathogen more sensitive to peroxide accumulation, compromising its ability to counteract the plant’s defence. These findings imply that the target genes associated with these enriched GO terms are important in response to pathogen infection, and the milRNAs that inhibit their target gene expression may play a role in preventing the activation of plant resistance or recognition. However, the functions of these target genes need to be verified further.

Our research about the infection-induced milRNAs in *Foc* provides a scientific theoretical foundation for exploring the function of this kind of milRNAs in fungi. It also supplies potential targets for the development of an efficient BFW control strategy, as well as the breeding of banana-resistant cultivars.

## Supplementary Material

Supplemental Material

Supplemental Material
