# Supplementary figures and images for: Unveiling microRNA-like small RNAs implicated in the initial infection of Fusarium oxysporum f. sp. cubense through small RNA sequencing
Source: Mycology. 2024 May 5;16(1):293–308. doi: 10.1080/21501203.2024.2345917 (PMC11899247; doi:10.1080/21501203.2024.2345917)

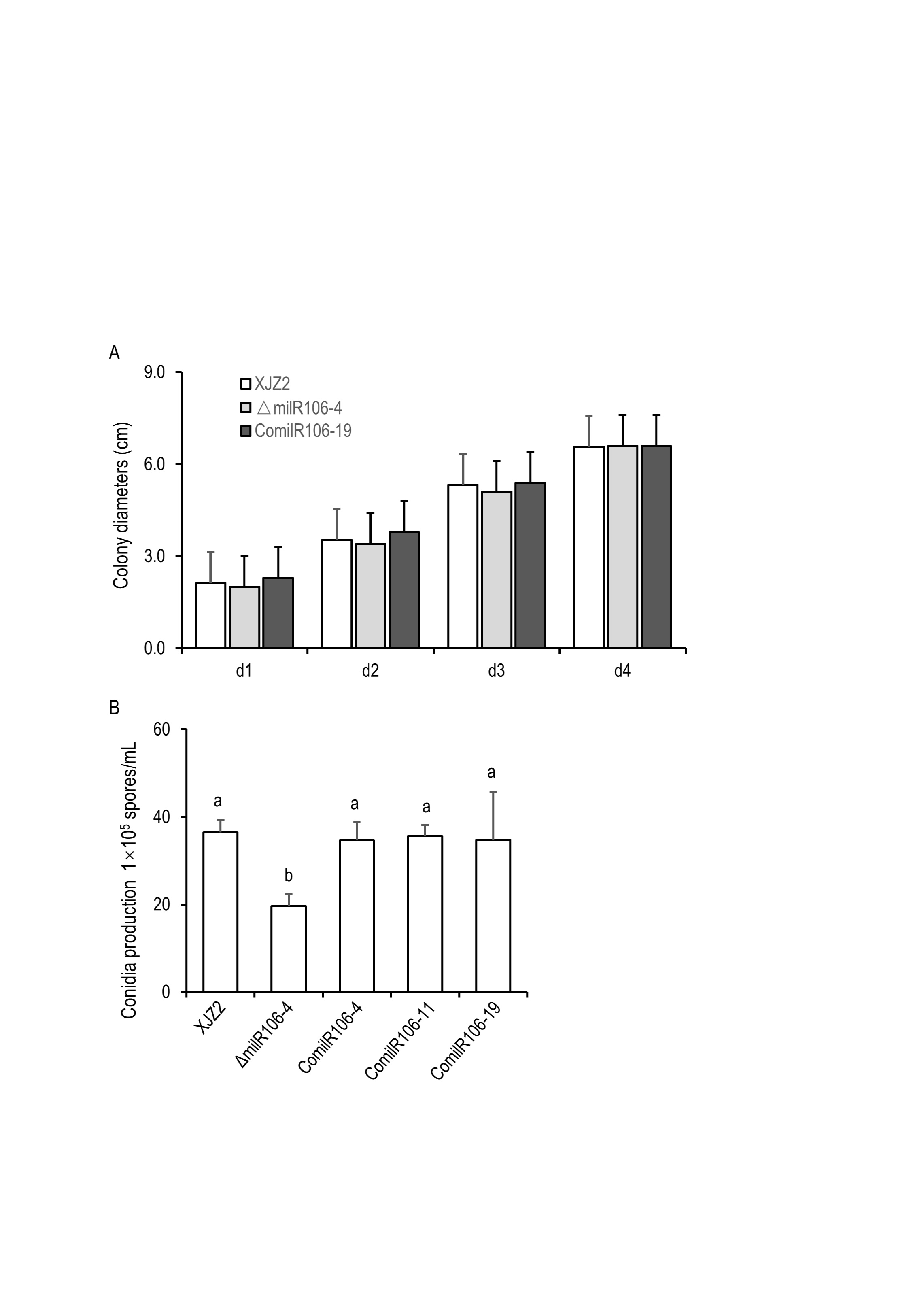

Supplement: Supplemental Material [file TMYC_A_2345917_SM3212.jpg]
